# Low adherence to CKD-specific dietary recommendations associates with impaired kidney function, dyslipidemia, and inflammation

**DOI:** 10.1038/s41430-020-00849-3

**Published:** 2021-02-02

**Authors:** Nadine Kaesler, Seema Baid-Agrawal, Sabine Grams, Jennifer Nadal, Matthias Schmid, Markus P. Schneider, Kai-Uwe Eckardt, Jürgen Floege, Manuela M. Bergmann, Georg Schlieper, Turgay Saritas

**Affiliations:** 1 Division of Nephrology and Clinical Immunology, University Hospital RWTH Aachen, Aachen, Germany; 2 Department of Nephrology and Transplant Center, Sahlgrenska University Hospital, University of Gothenburg, Gothenburg, Sweden; 3 German Institute of Human Nutrition Potsdam-Rehbruecke (DIfE), Nuthetal, Germany; 4 Institute for Medical Biometry, Informatics and Epidemiology, University Hospital Bonn, Bonn, Germany; 5 Department of Nephrology and Hypertension, Friedrich-Alexander-Universität Erlangen-Nürnberg, Erlangen, Germany; 6 Department of Nephrology and Hypertension, Klinikum Nürnberg, Paracelsus Private Medical University, Nürnberg, Germany; 7 Department of Nephrology and Medical Intensive Care Medicine, Charité Universitätsmedizin Berlin, Berlin, Germany; 8 Center for Nephrology, Hypertension, and Metabolic Diseases, Hannover, Germany

**Keywords:** Biomarkers, Kidney diseases, Nutrition

## Abstract

**Background/Objectives:**

A diet following chronic kidney disease (CKD)-specific recommendations is considered essential for optimal management of patients with CKD. However, data on the adherence to these recommendations and its implications for health-relevant biomarkers are lacking. The objectives were to estimate adherence to CKD-specific dietary recommendations, to identify characteristics and lifestyle variables associated with poor adherence, and to investigate the relationship of adherence with biomarkers.

**Methods:**

In this cross-sectional analysis, average dietary intake was estimated in 3193 participants with moderately severe CKD enrolled into the observational multicenter German CKD study using a food frequency questionnaire. A CKD diet score was developed to assess adherence to CKD-specific dietary recommendations based on intake of sodium, potassium, fiber, protein, sugar, and cholesterol. The associations of dietary adherence with characteristics, lifestyle variables, and biomarker levels were determined.

**Results:**

Logistic regression analysis revealed younger age, higher body mass index, male gender, lower educational attainment, various lifestyle variables (cigarette smoking, infrequent alcohol consumption, low physical activity), and lower estimated glomerular filtrate rate associated with lower adherence to dietary recommendations. Low adherence to dietary recommendations was further associated with dyslipidemia, higher uric acid, and C-reactive protein levels. Associations between low dietary adherence and biomarkers were mostly driven by low intake of fiber and potassium, and high intake of sugar and cholesterol.

**Conclusions:**

This study revealed differential characteristics and biomarkers associated with lower adherence to CKD-specific dietary recommendations. Promotion of CKD-specific dietary recommendations may help to mitigate the adverse prognosis in CKD patients.

## Introduction

Chronic kidney disease (CKD) is a globally rising health issue characterized by a high burden of comorbidities and mortality. Poor diet can increase the incidence and progression of CKD directly, e.g., through the path of obesity, or indirectly through its effects on contributing CKD risk factors such as diabetes or hypertension [1, 2]. Dietary interventions are highly relevant to reduce the risk of CKD complications such as sodium and volume overload, hyperkalemia, hyperphosphatemia, or malnutrition, which affect the cardiovascular system, bones, and other organs [2]. Nutritional education is therefore recognized as an important tool in the treatment of patients with CKD, but is underutilized as a preventive approach for slowing the progression of CKD [3].

Current recommendations for nutritional management of CKD typically focus on few individual components of diet such as protein and sodium [4, 5]. Data on adherence to dietary recommendations in patients with CKD are scarce [6], and it has not been shown whether adherence to dietary CKD recommendations associates with better kidney function or other biomarkers. We therefore assessed dietary and nutrient intake by a food frequency questionnaire (FFQ) in non-dialysis dependent CKD patients of the multicenter German Chronic Kidney Disease (GCKD) observational cohort study [7]. We developed a CKD diet score system based on the intake of sodium, potassium, fiber, total protein, sugar, and cholesterol to estimate the adherence to CKD-specific dietary recommendations from National Kidney Foundation Kidney Disease Outcomes Quality Initiative (NKF-KDOQI) and Kidney Disease: Improving Global Outcomes (KDIGO) [4, 5]. The objectives of the present study were to (a) estimate adherence to the CKD-specific dietary recommendations, (b) identify predictors for poor dietary adherence as defined by the CKD diet score system, and (c) associate health-relevant biomarkers with the degree of adherence to the CKD-specific dietary recommendations.

## Subjects and methods

Detailed information can be found in Supplementary Material.

### Study design

All participants of the study gave written consent and the ethics committees of all participating institutions approved the study. The study was conducted according to the guidelines laid down in the Declaration of Helsinki (6th version, 2008). The study was registered in the national registry of clinical studies (ID: DRKS00003971). The study design has been described previously in detail [8]. In brief, this prospective observational nationwide cohort study follows 5217 German patients with CKD of diverse etiology (Supplementary Fig. 1).

### Measurement of dietary intake

Average food intake was assessed using a self-administered FFQ of the European Prospective Investigation into Cancer and Nutrition (EPIC) Potsdam study, provided by the Human Study Center of the German Institute of Human Nutrition Potsdam-Rehbrücke. The reproducibility and validity of our questionnaire has previously been tested in the German EPIC cohort [9–11]. Out of 4754 approached participants who attended the GCKD follow-up study visit in year 2 (2012–2014), 3283 returned the FFQs (69.1%).

### Study population for present analysis

Participants who completed the FFQ with 26 or more missing answers were excluded from the analysis (*n* = 26) [12] as were those with an implausible energy intake to energy requirement ratio (1% top and bottom, *n* = 64) [13]. After all exclusions, the data set of analysis included 3193 participants. The participants included in our analysis had comparable characteristics to the total GCKD study population (Supplementary Table 1).

### Quality of dietary intake measured by the CKD score

To evaluate the quality of dietary intake among participants, we developed a score based on dietary recommendations from NKF-KDOQI and KDIGO (Supplementary Table 2). Consequently, we developed the score based on six dietary components, i.e., sodium, potassium, fiber, total protein, sugar, and cholesterol. Our score adjusts for energy intake using the density-based approach (amounts consumed per 1000 kcal) [14]. Due to the methodological features of a FFQ to rank participants according to their average dietary intake rather than measuring the actual intake [15, 16], we split dietary intake of each component into quintiles to score the adherence to it (Table 1) [15, 16]. For every component, the possible score ranges from one point (poorest adherence to recommendations) up to five points (highest adherence to recommendations). For adequacy components (i.e., potassium and fiber), higher intake of component result in higher scores. For moderation components (i.e., sodium, total protein, sugar, and cholesterol), lower intake of component result in higher scores. The scores of each component were summed up to calculate the overall CKD diet score = sodium score × 1.5 + potassium score + fiber score + protein score × 1.5 + sugar score + cholesterol score. As there is strong evidence that restriction of protein and sodium in patients with CKD has beneficial effects on kidney function, cardiovascular system, and quality of life [5], points for sodium and protein were multiplied by 1.5 to increase weight of these components for the calculation of the CKD diet score.

**Table 1:** CKD diet score system to estimate adherence to CKD-specific dietary recommendations applied in the German Chronic Kidney Disease (GCKD) observational cohort study (2012–2014).

Component	1 Point	2 Points	3 Points	4 Points	5 Points
Sodium/1000 kcal	>1.22 g	1.1–1.22 g	1.0–1.09 g	0.88–0.99 g	<0.88 g
Potassium/1000 kcal	<1.13 g	1.13–1.24 g	1.25–1.36 g	1.37–1.52 g	>1.53 g
Fiber/1000 kcal	<7.42 g	7.42–8.59 g	8.60–9.73 g	9.74–11.1 g	>11.1 g
Total protein/1000 kcal	>39.82 g	36.08–39.82 g	33.14–36.07 g	30.04–33.13 g	<30.04 g
Sugar/1000 kcal	>64.91 g	53.67–64.90 g	44.85–53.66 g	36.11–44.84 g	<36.11 g
Cholesterol/1000 kcal	>183.9 mg	164.73–183.9 mg	147.62–164.72 mg	127.95–147.61 mg	<127.95 mg

Dietary intake of each component was split into quintiles to score the adherence to the dietary components. Values describe range of dietary amount per 1000 kcal to achieve the respective score. The scores of each component were added to calculate the overall CKD diet score = sodium score × 1.5 + potassium score + fiber score + protein score × 1.5 + sugar score + cholesterol score. As dietary advice in CKD focuses mainly on restricting intake of dietary sodium and protein, points for sodium and protein were multiplied by 1.5 to increase weight of these components for the calculation of the CKD diet score. Thus, CKD diet score ranged from 7 points (poorest adherence to CKD-specific dietary recommendations) to 35 points (highest adherence to CKD-specific dietary recommendation).

Consequently, CKD diet score ranged from 7 to 35 points. The distribution of points calculated among participants is shown in Supplementary Fig. 2. Our dietary CKD score is a modification of previously validated dietary scores, i.e., Dietary Approaches to Stop Hypertension (DASH) diet and Mediterranean diet score, which was evident by a moderate to strong positive correlation of our score with these two scores (Supplementary Fig. 3 and Supplementary Table 3). The calculation of the DASH diet and Mediterranean diet score within our cohort has been shown earlier [13].

### Health-relevant biomarkers

A set of non-fasting biomarkers was determined by a central laboratory for routine clinical chemistry [8]. The biochemical data presented in this analysis resulted from the GCKD study follow-up visit in year 2, i.e., the period of 2012–2014, when data on dietary intake were collected [13].

### Statistical analysis

The CKD diet score was split into quintiles to investigate the distribution of participants using descriptive statistics. Continuous variables were expressed as means ± standard deviations or medians (interquartile range), if not normally distributed. Frequency of categorical variables was expressed in percentages. Normality of data was assessed by skewness and quantile–quantile plots. Pearson’s correlation coefficient was used to validate our score by measuring the strength of the association between CKD diet score, which has been newly developed, and the DASH diet or the Mediterranean diet score. Multivariate ordinal regression analysis was used to investigate the associations between a characteristic and adherence to CKD diet adjusting for all other co-variates in the model; the dependent variable was CKD diet score quintile in descending order. We analyzed whether means of health-related biomarkers adjusted for age, gender, body mass index (BMI), and smoking differed across CKD diet score quintiles applying generalized linear models. The geometric means are shown for the skewed variables UACR, triglyceride, HbA1c, and CRP. We tested whether the variations of adjusted means of health-related biomarkers across the score categories were statistically significant (*p* for trend) with the Wilcoxon-Rank-Sum test. Multivariable linear regression models were performed to evaluate the association of individual dietary components of the CKD diet scores and biomarkers. Two-sided *p* values < 0.05 were considered statistically significant and statistical analysis was performed with SPSS Version 26.

## Results

### Demographic participants' characteristics associated with adherence to CKD-specific dietary recommendations

Participants were stratified by quintiles based on CKD diet score. With the exception for dietary intake of sugar, which increased slightly with increasing CKD diet score, dietary intakes of sodium, potassium, fiber, total protein, and cholesterol were distributed across CKD diet score quintiles as expected by the scoring system (Supplementary Table 3). Energy intake seemed to be lower in participants with higher CKD diet score, but was overall similar between CKD diet score quintiles (Supplementary Table 3).

Compared to participants with higher CKD diet score, participants with a poor CKD diet score (i.e., less than 16 points) were younger, and more likely to be men, to have higher BMI, to be a smoker, to have a low educational attainment, to carry a diagnosis of diabetes mellitus, and to be prescribed medication for hypertension, diabetes, hyperlipidemia, or gout (Table 2). Participants with a low CKD diet score also had a lower self-reported use of alcoholic beverages and low levels of physical activity.

**Table 2:** Characteristics of participants with chronic kidney disease participating in the German Chronic Kidney Disease (GCKD) observational cohort study (2012–2014).

Characteristics	CKD diet score quintiles (score of)					
	Q1 (≤15) *N* = 826	Q2 (16–17) *N* = 605	Q3 (18–19) *N* = 657	Q4 (20–21) *N* = 532	Q5 (≥22) *N* = 573	*P* value
Age, years, mean ± SD	62 ± 12.5	63.3 ± 11.3	63.9 ± 11.7	63.9 ± 11.3	65.2 ± 10.8	**<0.001**
Women (%)	26.5	41	45.2	43.3	56.9	**<0.001**
BMI (kg/m^2^), median (IQR)	29.1 (7.5)	28.1 (6.6)	27.6 (6.4)	27.8 (7.6)	27.4 (7)	**<0.001**
Smoking (%)						**<0.001**
Smoker	20.3	15.1	12.7	10.5	11.6	
Former smoker	44.4	39.9	41.9	42.9	41.6	
Non-smoker	35.2	45.0	45.4	46.5	46.8	
Alcohol (%)						**<0.001**
≥3×/week	14.9	17.6	20.7	24.1	20.6	
<1–2×/week	85.1	82.4	79.3	75.9	79.4	
German school education (%)						**<0.001**
≤9th grade	56.2	51.7	51.3	47.2	48.1	
10th grade	28.1	31.0	30.1	32.2	29.3	
≥12th grade	15.7	17.3	18.6	20.6	22.6	
Physical activity for 30 min (%)						**0.001**
<1×/week	16.8	15.9	14.6	12.5	9.3	
1–2×/week	26.1	25.7	25.2	26.0	27.3	
3–5×/week	29.4	29.7	30.0	31.7	31.2	
>5×/week	27.7	28.7	30.2	29.8	32.1	
SBP, mmHg, mean ± SD	140 ± 20.1	138.9 ± 19.3	138.6 ± 18.8	138.2 ± 19.4	139.1 ± 20	0.137
DBP, mmHg, mean ± SD	79.8 ± 11.8	79.6 ± 11.5	79.2 ± 10.5	79.6 ± 11.7	79.3 ± 11.5	0.494
Diabetes mellitus (%)	34.5	30.7	31.7	30.8	26.9	**0.006**
Anti-hypertensive medication (%)	92.7	92.4	91.0	92.9	90.6	0.230
Anti-diabetic medication (%)	26.6	23.5	25.3	24.4	22.0	0.099
Lipid-lowering medication (%)	50.4	52.4	49.3	49.4	49.4	0.471
Anti-gout medication (%)	37	32.6	28.2	31.8	28.6	**0.001**

*P* values reflect significance of a linear trend across quintiles of CKD diet score, using one-way ANOVA, *χ*^2^, or Jonckheere–Terpstra tests, where appropriate.

Values are expressed as mean values ± standard deviation, medians (interquartile ranges (IRQ)) or numbers (percentages), as appropriate. Bold *P* values indicate statistical significance.

*BMI* body mass index, *DBP* diastolic blood pressure, *SBP* systolic blood pressure, *Q* quintile.

### Characteristics associated with adherence to CKD-specific dietary recommendations

Table 3 depicts the multivariable adjusted odds ratios for participants characteristics associated with a poor adherence to the CKD-specific dietary recommendations. Relevant characteristics associated with lower CKD diet score quintiles were younger age, higher BMI, male sex, smoking (vs. non-smoker), less frequent consumption of alcoholic beverages, low physical activity (<1×/week vs. >5×/week), low educational attainment, and lower eGFR. No association was found between adherence to the CKD-specific dietary recommendations and diabetes mellitus, albuminuria or intake of medication for hypertension, hyperlipidemia, or gout.

**Table 3:** Associations between adherence to CKD-specific dietary recommendations and characteristics of participants of the German Chronic Kidney Disease (GCKD) observational cohort study 2012–2014 as obtained from multivariable ordinal regression.

Effect	OR^a^ (95 % CI)
Age (per 1-SD increase)	0.78 (0.72, 0.85)
BMI (per 1-SD increase)	1.14 (1.06, 1.23)
Gender (male vs. female)	2.18 (1.86, 2.55)
Smoking (vs. non-smoker)	
Smoker	1.42 (1.13, 1.77)
Former smoker	0.95 (0.82, 1.12)
Alcohol (≥3×/week vs. <1–2×/week)	0.66 (0.55, 0.79)
Physical activity for 30 min (vs. >5×/week)	
<1×/week	1.48 (1.17, 1.87)
1–2×/week	1.05 (0.87, 1.27)
3–5×/week	1.11 (0.93, 1.33)
German school education (vs. ≥12th grade)	
≤9th grade	1.51 (1.24, 1.85)
10th grade	1.32 (1.07, 1.63)
Diabetes mellitus (yes vs. no)	1.04 (0.88, 1.23)
eGFR (per 1-SD increase)	0.92 (0.85, 1.0)
UACR (per 1-SD increase)	1.02 (0.94, 1.1)
Intake of lipid-lowering medication	1.01 (0.87, 1.17)
Intake of anti-hypertensive medication	0.93 (0.71, 1.22)
Intake of anti-gout medication	1.09 (0.93, 1.28)

Dependent variable: CKD diet score quintile, descending order.

*BMI* body mass index, *eGFR* estimated glomerular filtration rate, *UACR* urine albumin-to-creatinine ratio, *SD* standard deviation.

^a^Mutually adjusted odds ratio using ordinal logistic regression.

### Association of health-relevant biomarkers with adherence to the CKD-specific dietary recommendations

Table 4 shows health-relevant biomarkers adjusted for age, gender, BMI, and smoking in participants across quintiles of CKD diet score. Participants with higher quintile of CKD diet score had lower serum urea, total cholesterol, LDL-cholesterol, triglyceride, uric acid, phosphate and CRP levels, and higher HDL-cholesterol levels. Further adjustments for physical activity resulted in very similar mean values (data not shown). The unadjusted biomarker values are shown in Supplementary Table 4.

**Table 4:** Means adjusted for age, gender, body mass index, and smoking of health-relevant biomarkers across degrees of adherence to the CKD-specific dietary recommendations of participants of the German Chronic Kidney Disease (GCKD) observational cohort study (2012–2014).

Lab parameters	CKD diet score quintiles (score of)					
	Q1 (≤15) *N* = 826	Q2 (16–17) *N* = 605	Q3 (18–19) *N* = 657	Q4 (20–21) *N* = 532	Q5 (≥22) *N* = 573	*P* value
eGFR (mL/min/1.73m^2^), mean ± SD	46.9 ± 19.5	48.0 ± 18.6	47.9 ± 17.7	49.1 ± 17.9	50.5 ± 19.1	0.262
UACR (mg/g), g.mean ± SD	55.1 ± 8.4	54.3 ± 8.0	57.1 ± 8.1	48.2 ± 7.8	54.0 ± 7.9	0.068
Urea (mg/dL), mean ± SD	28.6 ± 12.9	27.7 ± 11.6	27.9 ± 11.2	27.2 ± 11.1	26.5 ± 10.8	**0.001**
Albumin (g/L), mean ± SD	40.7 ± 4.6	40.9 ± 4.1	41.1 ± 4.2	41.4 ± 4.3	41.1 ± 4.2	0.104
Cholesterol (mg/dL), mean ± SD	213.8 ± 49.9	211.8 ± 51.1	216.6 ± 47.6	211.0 ± 50.1	210.3 ± 48.4	**0.007**
HDL-cholesterol (mg/dL), mean ± SD	55.4 ± 17.2	56.0 ± 17.2	58.6 ± 19.8	58.2 ± 19.2	60.0 ± 18.9	**<0.001**
LDL-cholesterol (mg/dL), mean ± SD	123.0 ± 43.2	120.3 ± 44.2	124.3 ± 40.1	119.6 ± 41.5	120.3 ± 40.9	**0.040**
Triglyceride (mg/dL), g.mean ± SD	177.3 ± 1.8	178.2 ± 1.7	175.5 ± 1.7	171.9 ± 1.7	159.0 ± 1.7	**<0.001**
Uric acid (mg/dL), mean ± SD	7.2 ± 1.9	7.1 ± 1.7	7.2 ± 1.8	7.1 ± 1.7	7.0 ± 1.8	**0.003**
Phosphorus (mmol/L), mean ± SD	1.2 ± 0.3	1.2 ± 0.2	1.2 ± 0.2	1.2 ± 0.2	1.1 ± 0.2	**0.028**
Calcium (mmol/L), mean ± SD	2.5 ± 0.2	2.5 ± 0.1	2.5 ± 0.2	2.5 ± 0.2	2.5 ± 0.2	0.332
HbA1c (%), g.mean ± SD	6.1 ± 1.2	6.0 ± 1.1	6.1 ± 1.1	6.1 ± 1.1	6.0 ± 1.1	0.755
CRP (mg/L), g.mean ± SD	2.4 ± 3.2	2.2 ± 3.1	2.2 ± 3.1	2.1 ± 3.2	1.9 ± 3.2	**<0.001**

Values are expressed as mean values ± standard deviation adjusted for age, gender, body mass index, and smoking. The geometric means (g.mean) are shown for the highly skewed variables UACR, triglycerides, HbA1c, and CRP.

Conversion factors for units: calcium in mmol/L to mg/dL, ×4; phosphorus (inorganic) in mmol/L to mg/dL. Bold *P* values indicate statistical significance.

*eGFR* estimated glomerular filtration rate based on creatinin (scr), *HbA1c* glycated hemoglobin, *HDL-cholesterol* high-density lipoprotein-cholesterol, *LDL-cholesterol* low-density lipoprotein-cholesterol, *UACR* urine albumin-to-creatinine ratio, *CRP* C-reactive protein, *Q* quintile.

### Association between the individual components of the CKD diet score and biomarkers

Of the individual components of the CKD diet score, higher intake of potassium and fiber was associated with higher eGFR, while higher intake of sodium, total protein, sugar, and cholesterol trended to be associated with lower eGFR (Table 5). Higher intake of fiber was associated with lower serum CRP values, and higher intake of cholesterol was associated with a higher CRP values (Table 5). We further observed that lower intake of sugar and cholesterol was associated with higher serum HDL-cholesterol (Table 6). Higher intake of cholesterol and lower intake of sodium were associated with higher LDL-cholesterol (Table 6). Lower intake of fiber and higher intake of sugar were associated with higher triglyceride levels (Table 6). None of the CKD diet score components were individually associated with albuminuria and serum albumin (Supplementary Table 5). Although higher CKD diet score associated with lower uric acid (Table 4), none of the components were individually driving this association (Supplementary Table 5).

**Table 5:** Multivariable adjusted linear regression models for explanation of eGFR and Ln CRP by individual components of CKD diet score among participants of the German Chronic Kidney Disease (GCKD) observational cohort study (2012–2014).

CKD diet score components^a^	eGFR			Ln CRP		
	β-coefficient	SE	*P* value	β-coefficient	SE	*P* value
Sodium (per 1-SD increase)	−0.284	0.443	0.522	−0.035	0.025	0.156
Potassium (per 1-SD increase)	1.465	0.649	**0.024**	−0.010	0.036	0.776
Fiber (per 1-SD increase)	1.206	0.491	**0.014**	−0.100	0.028	**<0.001**
Total protein (per 1-SD increase)	−0.855	0.639	0.181	0.025	0.036	0.480
Sugar (per 1-SD increase)	−0.469	0.452	0.300	−0.001	0.025	0.969
Cholesterol (per 1-SD increase)	−0.760	0.558	0.173	0.097	0.032	**0.002**

*BMI* body mass index, *eGFR* estimated glomerular filtration rate, *CRP* C-reactive protein, *SD* standard deviation.

^a^Each component of CKD diet score was added individually into the model (not adjusted for the other food components), adjusting for gender, age, BMI, caloric intake, smoking, alcohol consumption, school education, and physical activity. CRP concentrations were log transformed due to skewed data, and one unit was added prior to log transformation to avoid loss of data when log(0) is rendered as missing.

Bold *P* values indicate statistical significance.

**Table 6:** Multivariable adjusted linear regression models for explanation of HDL, LDL, or triglycerides by individual components of CKD diet score per 1-SD increase among participants of the German Chronic Kidney Disease (GCKD) observational cohort study (2012–2014).

CKD diet score components^a^	HDL-cholesterol			LDL-cholesterol			Ln Triglyceride		
	β-coefficient	SE	*P* value	β-coefficient	SE	*P* value	β-coefficient	SE	*P* value
Sodium	0.143	0.408	0.727	−3.743	1.044	**<0.001**	−0.015	0.013	0.260
Potassium	0.220	0.611	0.719	1.378	1.567	0.379	−0.003	0.020	0.860
Fiber	0.769	0.464	0.098	−2.037	1.190	0.087	−0.031	0.015	**0.037**
Total protein	−0.790	0.605	0.192	0.673	1.554	0.655	0.018	0.019	0.353
Sugar	−1.628	0.428	**<0.001**	−1.055	1.102	0.339	0.031	0.014	**0.025**
Cholesterol	−1.236	0.532	**0.020**	3.248	1.364	**0.017**	0.025	0.017	0.148

*BMI* body mass index, *HDL-cholesterol* high-density lipoprotein-cholesterol, *LDL-cholesterol* low-density lipoprotein-cholesterol, *SD* standard deviation.

^a^Each component of CKD diet score was added individually into the model (not adjusted for the other food components), adjusting for gender, age, BMI, caloric intake, smoking, alcohol consumption, school education, and physical activity. The variable serum triglyceride was log transformed due to skewed data. Bold *P* values indicate statistical significance.

## Discussion

Our data suggest that adherence to CKD-specific dietary recommendations is mostly driven by gender, age, lifestyle, or socioeconomic factors rather than the prevalence of comorbidities such as hypertension, diabetes, hyperlipidemia, and gout. Our results also show that lower adherence to CKD-specific dietary recommendations associates with lower kidney function, “bad” lipid profile, higher inflammation, and higher uric acid values, all factors known to be associated with higher risk for cardiovascular events.

Several studies in non-CKD populations have related “unhealthy” diet with physical inactivity, smoking, younger age, and lower socioeconomic status [17–19]. Adopting new, healthy lifestyle and dietary habits can be very difficult. The Prospective Urban Rural Epidemiology (PURE) study has shown that only a small proportion of individuals were following a “healthy” diet even after suffering a coronary heart disease or stroke event [20]. Similar to the PURE study, we could not find any association between prevalence of comorbidities and adherence to dietary recommendations, underscoring the large gap between actual and recommended dietary intake in CKD patients with additional comorbidities. Our finding that infrequent or no alcohol consumption was associated with low CKD diet score should be interpreted with caution. A possible explanation could be that the consumption frequency may decrease with a worsening of health conditions, as the category of infrequent alcohol consumption may also include the “sick quitter.” Furthermore, infrequent alcohol use may be associated with heavy drinking occasions, whereas regular alcohol use is often associated with lower consumption per occasion, depicting a detrimental lifestyle factor [21].

Higher dietary intake of potassium and fiber was associated with better kidney function based on eGFR. The guidelines for CKD patients with dyslipidemia endorse a dietary fiber intake of 20–30 g per day [22]. Furthermore, the KDOQI guideline suggests a potassium intake between 2 and 4 g/d for CKD stage 3 and 4. There is growing evidence on the beneficial effects of dietary fiber and potassium for kidney function by reducing secretion of pro-inflammatory and pro-atherogenic cytokines [23–26]. This is consistent with our observation of inverse correlation between fiber intake and CRP values. Several studies have shown that adherence to “healthy” dietary patterns (e.g. Mediterranean diet) rich in fiber and potassium associated with reduced risk for CKD incidence, progression, and kidney failure [27, 28]. Fruits and vegetables, which are important sources of fiber and potassium, are often restricted in CKD due to concerns of hyperkalemia, although hyperkalemia is rarely seen in early stages of CKD. Thus, future CKD nutrition guidelines should more clearly encourage patients with mild- to moderate CKD (i.e., CKD stage 1–3) to increase dietary intake of fiber and potassium.

Although the CKD guidelines give most attention to restriction of dietary intake of sodium and total protein, none of the health-relevant biomarkers was found to be associated with the intake of total protein or sodium, except for LDL cholesterol, which decreased with higher intake of sodium. The inverse association between LDL and sodium appears surprising, but a Cochrane meta-analysis showed a similar inverse association in which greater dietary sodium reduction resulted in increase of plasma cholesterol and triglyceride [29, 30]. Dietary sodium restriction helps to control volume status and hypertension, but it is controversial whether sodium restriction can slow down CKD progression, as controlled studies are lacking [31]. Evidence from animal studies suggests that high protein diet causes glomerular hyperfiltration, which is known to cause kidney damage. In humans, high intake of protein was associated with the risk for CKD incidence and progression [32]. However, the net effect of protein intake on human kidney is less clear, as the amount of other potential beneficial food components (e.g. fiber) could be low in high protein diets.

For patients with CKD, the recommended intake of cholesterol is 200–300 mg or less per day [22]. We found that higher intake of cholesterol correlated with higher CRP and LDL, but lower HDL-cholesterol, which is consistent with previous reports [33, 34]. There are no randomized trials that have assessed the efficacy of a low fat, low cholesterol diet in CKD patients. However, evidence from several studies, including those examining dietary patterns in CKD, suggest that a so-called Mediterranean diet, which is low in saturated fat and cholesterol improves health [35–37].

It has been shown that not only high intake of saturated fats, but also increased sugar intake by sugar-sweetened beverages associates with dyslipidemia [38]. Higher intake of sugar was associated with lower HDL-cholesterol and higher triglyceride levels. KDOQI recommends a restriction of sugar to less than 10% of energy intake [39]. High consumption of sugar has been demonstrated to associate with incidence of CKD in the general population [40]. However, no association was found between sugar intake and eGFR decline in participants with preexistent CKD from the Multi-Ethnic Study of Atherosclerosis study [41]. Similarly, we also did not observe an association between sugar intake and eGFR.

Our study has several strengths. The large sample size, based on a comparatively high FFQ response rate in one of the largest CKD cohort worldwide, is a strength of this study. Furthermore, standardized questionnaires to assess participants’ characteristics including risk factors and comorbidities were completed during an in-person study visits conducted by trained study nurses. In addition, all laboratory parameters were measured in centralized laboratories to reduce measurement bias due to multiple centers.

There are also some limitations of the study. Dietary intake may differ in the 30% of study participants who did not return the questionnaire, although the participants included in our analysis had similar demographic and clinical characteristics compared with the total GCKD study population. The German CKD population limits generalizability to diseased cohorts in other settings as well as other geographical regions. The self-administered FFQ is a widely applied instrument in large studies because of its easy applicability and high compliance of the participants [15]; however, since this FFQ has not been developed specifically for population with CKD, some food groups may have been over- or underreported because of CKD-related dietary restriction.

Our study implies for future studies to focus more on patient-centered behavioral interventions to promote implementation of beneficial dietary patterns in daily life. In addition, large-scale clinical trials investigating the effects of dietary patterns consistent with CKD-specific dietary recommendations on clinical outcomes are needed. To the best of our knowledge, we have, for the first time, assessed the adherence to CKD-specific dietary recommendations and its association with patient’s characteristic and biomarkers systemically within a large CKD cohort. Our data emphasize the need for better promotion of an individualized and proactive dietary intervention especially in men with prevalent adverse lifestyle factors such as high BMI or smoking, as these are factors responsible for poor adherence to CKD-specific dietary recommendations. Our data support the CKD-specific dietary recommendations in order to provide better treatment to CKD patients, as adherence to these recommendations was associated with better kidney function, healthier lipid profile, lower inflammation, and lower uric acid levels. Rather than focusing mainly on restriction of dietary sodium and protein, we believe that CKD guidelines should comment more prominently on the role of dietary fiber, potassium, cholesterol, and sugar intake, as the associations were mainly driven by the adequate intake of these components.

## Supplementary information

Supplemental Material

## Data Availability

Public posting of individual level data is not covered by the informed consent form signed by each patient participating in this study. As stated in the patient consent form and approved by the Ethics Committees, a dataset containing pseudonyms can be obtained by collaborating scientists upon approval of a scientific project proposal.
