# Supplementary material for: Low adherence to CKD-specific dietary recommendations associates with impaired kidney function, dyslipidemia, and inflammation
Source: Eur J Clin Nutr. 2021 Feb 2;75(9):1389–97. doi: 10.1038/s41430-020-00849-3 (PMC8416654; doi:10.1038/s41430-020-00849-3)
Supplement: Supplementary file 1 — Supplemental Material [file 41430_2020_849_MOESM1_ESM.pdf]

Supplementary Material

Title: Low adherence to CKD-specific dietary recommendations associates with impaired kidney function, dyslipidemia and inflammation.

Authors: Nadine Kaesler, Seema Baid-Agrawal, Sabine Grams, Jennifer Nadal, Matthias Schmid, Markus P. Schneider, Kai-Uwe Eckardt, Jürgen Floege, Manuela M. Bergmann, Georg Schlieper, Turgay Saritas.

Supplementary Figure 1: Flow chart of participants of the German Chronic Kidney Disease (GCKD) observational cohort study (2012-2014) included in the analysis.

Supplementary Figure 2: Distributions of CKD diet score within the German Chronic Kidney Disease (GCKD) observational cohort study (2012-2014).

Supplemental Figure 3: Correlation between CKD diet score and the DASH diet (A) or Mediterranean diet score (B).

Supplementary Table 1: Baseline characteristics of participants included in study and all German Chronic Kidney Disease (GCKD) participants.

Supplementary Table 2. CKD-specific dietary recommendations as published by the National Kidney Foundation Kidney Disease Outcomes Quality Initiative (NKF-KDOQI) and Kidney Disease: Improving Global Outcomes (KDIGO).

Supplementary Table 3. Dietary intake by CKD diet score quintiles among participants of the German Chronic Kidney Disease (GCKD) observational cohort study (2012-2014).

Supplementary Table 4. Health-relevant biomarkers across degrees of adherence to the CKD-specific dietary recommendations of participants of the German Chronic Kidney Disease (GCKD) observational cohort study (2012-2014).

Supplementary Table 5. Multivariable adjusted linear regression models for explanation of log-UACR, serum albumin and uric acid by individual components of CKD diet score per 1-SD increase among participants of the German Chronic Kidney Disease (GCKD) observational cohort study (2012-2014).

Supplementary Methods and References

Supplementary Figure 1. Flow chart of participants of the German Chronic Kidney Disease (GCKD) observational cohort study (2012-2014) included in the analysis.

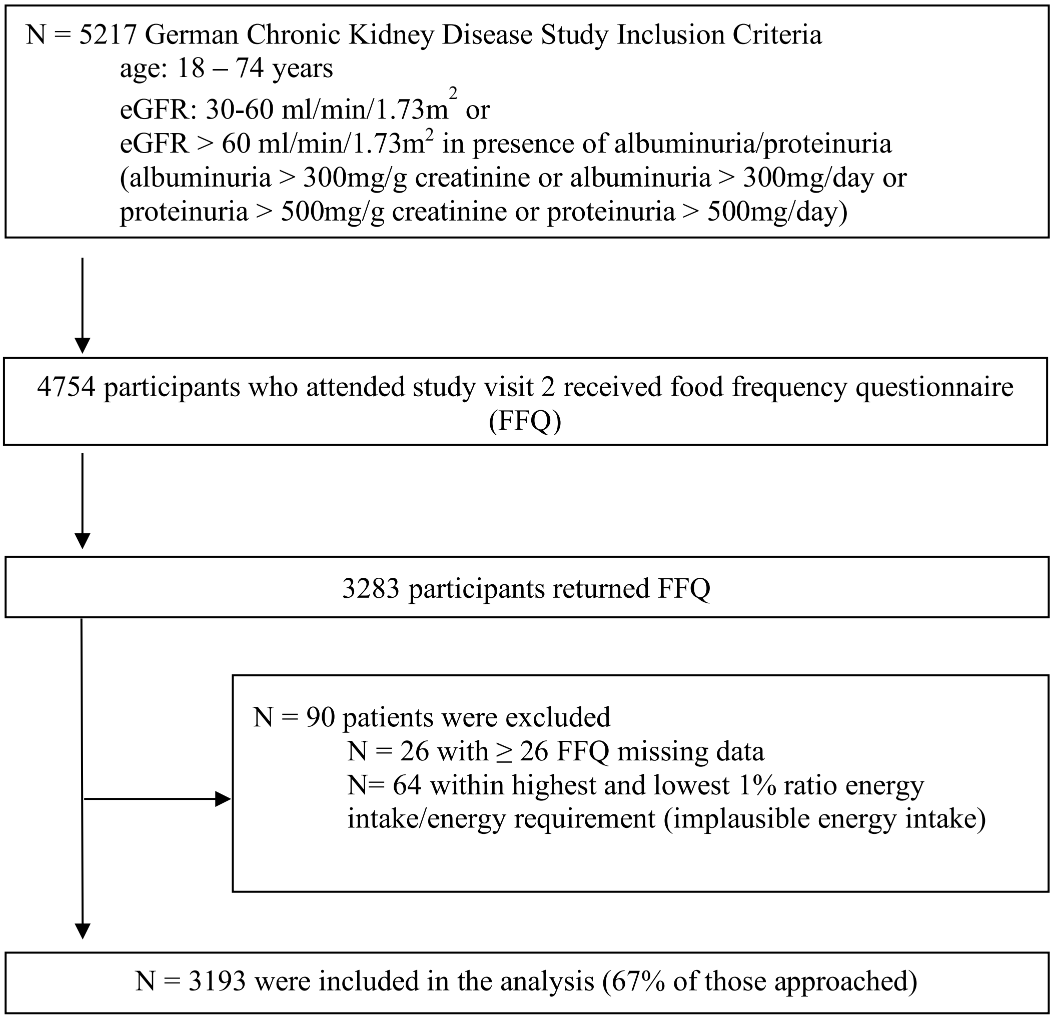

Supplementary Figure 2: Distributions of CKD diet score within the German Chronic Kidney Disease (GCKD) observational cohort study (2012-2014). Mean value: 18.0 ± 3.6; N = 3193.

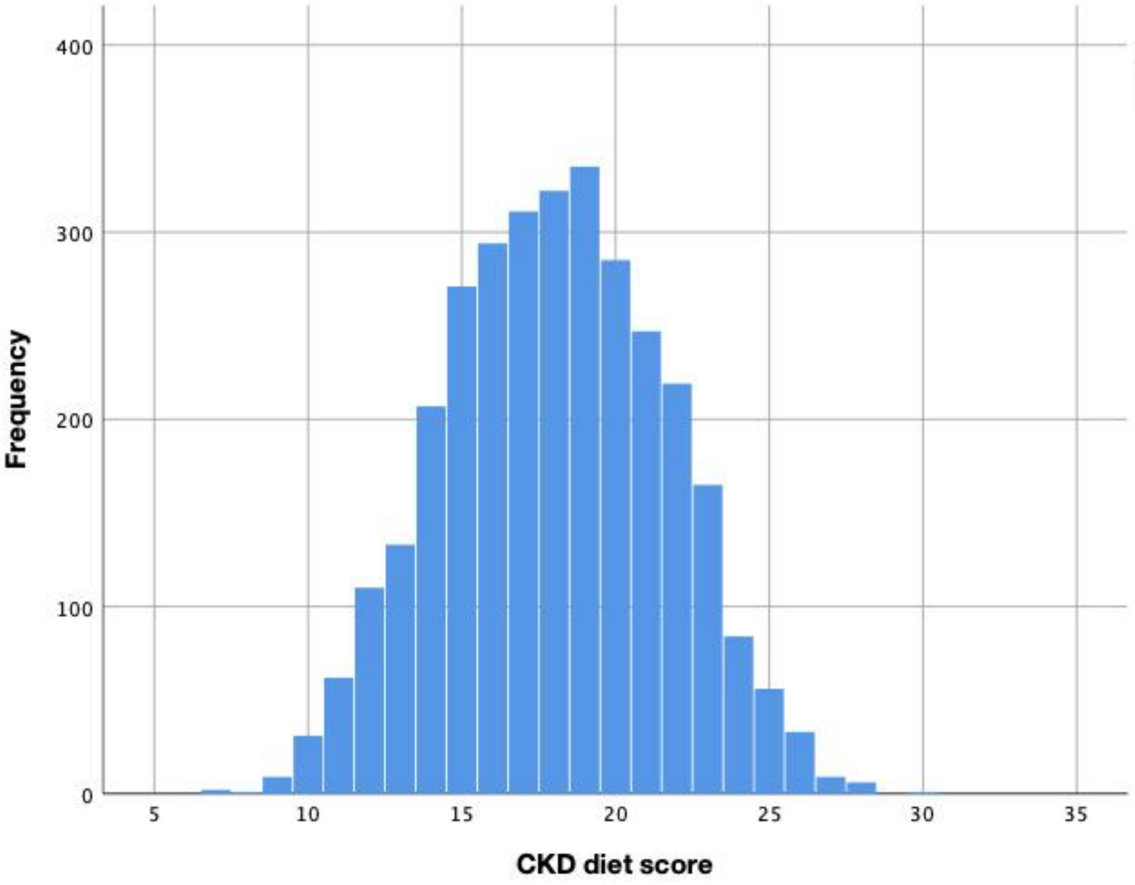

Supplemental Figure 3: Correlation between CKD diet score and the DASH diet (A) or Mediterranean diet score (B).

A

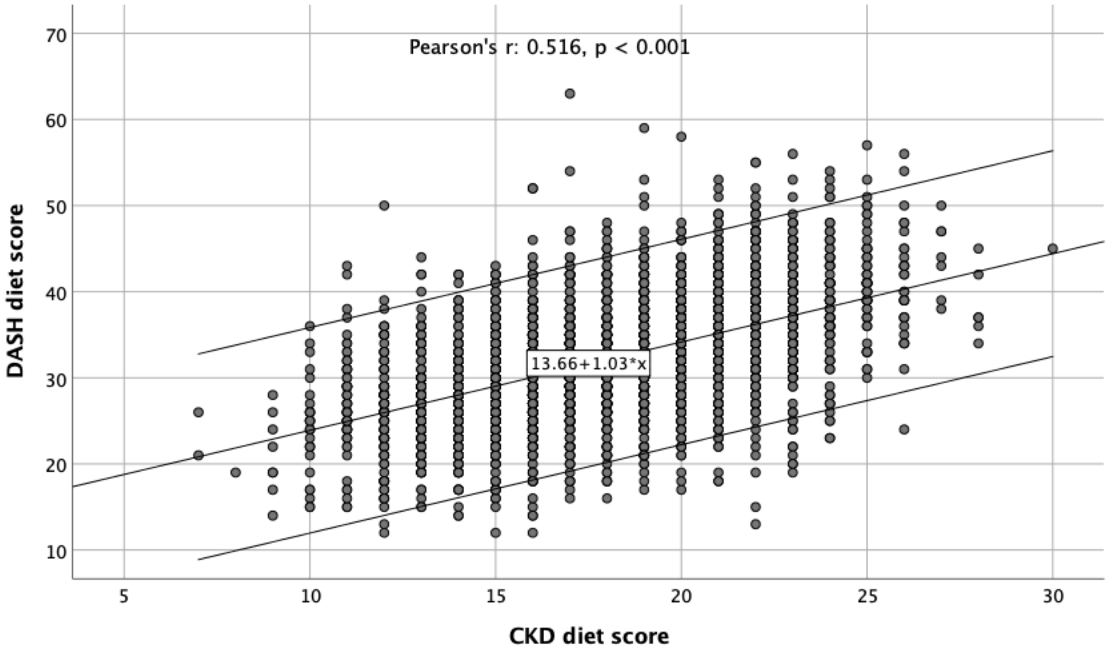

B

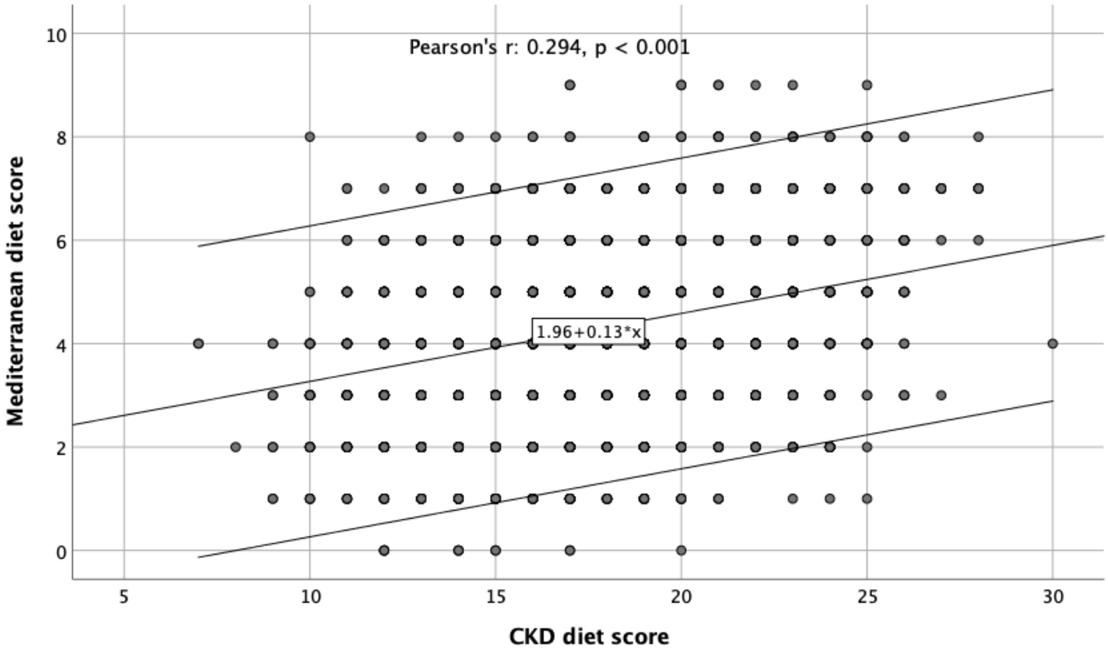

Supplementary Table 1: Baseline characteristics of participants included in study and all German Chronic Kidney Disease (GCKD) participants.

Baseline Characteristics	Included N = 3193	Total N = 5127
Age, years, mean ± SD	63.5 ± 11.6	63.1 ± 12
Women (%)	41.4	40.0
BMI, (kg/m^2), median (IQR)	28.0 (6.9)	28.9 (7.5)
Smoking (%)		
Smoker	14.6	15.9
Former smoker	42.3	43.1
Non-Smoker	43.1	41.0
Alcohol consumption (%)		
≥3x/week	19.2	18.9
<1-2x/week	80.8	80.5
German school education (%)		
≤9th grade	51.4	53.3
10th grade	30.0	28.0
≥12th grade	18.6	16.7
Physical activity for 30 minutes (%)		
<1×/week	14.1	16.2
1-2×/week	26.1	25.3
3-5×/week	30.3	28.2
>5×/week	29.5	29.0
Systolic blood pressure, mmHg, mean ± SD	139.0 ± 19.5	138.3 ± 21.1
Diabetes mellitus (%)	31.2	35.8
Hyperlipidemia (%)	50.2	51.2
eGFR, (ml/min/1.73m^2), mean ± SD	48.3 ± 18.6	47.7 ± 19.2
UACR, (mg/g), median (IQR)	44.9 (290.4)	49.8 (315.1)
Urea, (mg/dL), mean ± SD	27.7 ± 11.7	28.4 ± 12.3
Serum albumin, (g/L), mean ± SD	41.0 ± 4.3	40.9 ± 4.5
Serum-cholesterol, (mg/dL), mean ± SD	212.9 ± 49.4	212.1 ± 50.9
Serum HDL cholesterol, (mg/dL), mean ± SD	57.5 ± 18.7	57 ± 18.7
Serum LDL cholesterol, (mg/dL), mean ± SD	121.7 ± 42.1	121.2 ± 42.7
Serum triglycerides, (mg/dL), mean ± SD	201.8 ± 131.4	202.6 ± 138.9
Serum total calcium, (mmol/L), median (IQR)	2.5 (0.2)	2.5 (0.2)
Serum phosphorus (inorganic), (mmol/L), median (IQR)	1.1 (0.3)	1.2 (0.3)
Serum uric acid, (mg/dL), mean ± SD	7.1 ± 1.8	7.2 ± 1.9
CRP, (mg/L), median (IQR)	2.1 (3.7)	2.3 (4)
HbA1c, median (IQR)	5.8 (0.7)	5.9 (0.9)
Anti-hypertensive medication (%)	92	92.4
Anti-diabetic medication (%)	24.6	28.5
Lipid-lowering medication (%)	50.2	51.2
Anti-gout medication (%)	32	32.8

Abbreviations: BMI, body mass index; eGFR, estimated glomerular filtration rate (CKD-EPI equation); UACR, urine albumin-to-creatinine ratio; HDL, high-density lipoprotein; LDL, low-density lipoprotein; HbA1c, glycated hemoglobin.

Supplementary Table 2. CKD-specific dietary recommendations as published by the National Kidney Foundation Kidney Disease Outcomes Quality Initiative (NKF-KDOQI) and Kidney Disease: Improving Global Outcomes (KDIGO).

**Recommended intake per day**	
Sodium (g)	< 2.3
Potassium (g)	2 – 4
Fiber (g)	> 20
Protein (g/kg ideal body weight)	0.55 - 0.9
Sugar (% of total energy)	<10
Cholesterol (mg)	< 200 - 300

Supplementary Table 3. Dietary intake by CKD diet score quintiles among participants of the German Chronic Kidney Disease (GCKD) observational cohort study (2012-2014).

	**CKD diet score quintiles (score of)**				
**Characteristic**<br>median (IQR)	**Q1**<br>**(≤15)**<br>**N = 826**	**Q2**<br>**(16-17)**<br>**N = 605**	**Q3**<br>**(18-19)**<br>**N = 657**	**Q4**<br>**(20-21)**<br>**N = 532**	**Q5**<br>**(≥22)**<br>**N = 573**
Sodium intake (g/day)	2.4 (1.3)	2.3 (1.2)	2.2 (1.2)	2.2 (1.1)	1.8 (0.8)
Potassium intake (g/day)	2.7 (1.2)	2.8 (1.2)	2.8 (1.2)	2.8 (1.2)	2.9 (1.3)
Dietary fiber (g/day)	16.9 (8.6)	18.8 (9.5)	20.1 (9)	21.4 (9.6)	21.9 (9.4)
Total protein (g/day)	87.4 (46.9)	78 (37.1)	72.7 (34.7)	68.4 (29.1)	60.4 (27.9)
Total protein per IBW (g/day)	1.3 (0.7)	1.2 (0.5)	1.1 (0.5)	1.1 (0.4)	1.0 (0.4)
Plant-based protein (g/day)	22.9 (12.3)	23.9 (13.6)	24.7 (13.3)	26.3 (13.7)	24.7 (12.4)
Fat (g/day)	104.3 (54.1)	95.2 (50.5)	94.2 (47.5)	90.6 (42)	85.2 (42.7)
Cholesterol (mg/day)	411.2 (192.9)	356.2 (164.6)	328.5 (144.3)	291.8 (134)	247.7 (118.7)
Poly-unsaturated fats (g/day)	15.6 (9.2)	14.9 (8.8)	14.9 (9.2)	15.3 (9.3)	15.1 (9.3)
Saturated fats (g/day)	41.8 (21.6)	37.6 (20.1)	37.2 (18.7)	34.8 (16.7)	30.5 (14.9)
Sugar (g/day)	98.7 (61.9)	99.4 (67)	105.2 (69)	107.2 (68.9)	109.2 (70.8)
Carbohydrates (g/day)	207.7 (108)	210.8 (118.9)	228.2 (125)	235.7 (121.1)	221.8 (110.7)
Energy intake per IBW (kcal/day)	33 (16.7)	33.3 (15.7)	34.3 (14.8)	34.1 (13.5)	33 (15.1)
Energy Intake (kcal/day)	2205.7 (1096.2)	2145.3 (1094.9)	2159.1 (1043.6)	2181.8 (1014)	2034.7 (987.2)
DASH diet score, mean ± SD	27.6 ± 5.8	30.7 ± 6.0	32.2 ± 6.1	34.5 ± 6.3	37.8 ± 6.9
Mediterranean diet score, mean ± SD	3.8 ± 1.6	4.2 ± 1.6	4.3 ± 1.5	4.7 ± 1.5	5.0 ± 1.5

Abbreviations: IBW, ideal body weight.

Supplementary Table 4. Health-relevant biomarkers across degrees of adherence to the CKD-specific dietary recommendations of participants of the German Chronic Kidney Disease (GCKD) observational cohort study (2012-2014).

	CKD diet score quintiles (score of)					
**Characteristic**	**Q1 (≤15) N = 826**	**Q2 (16-17) N = 605**	**Q3 (18-19) N = 657**	**Q4 (20-21) N = 532**	**Q5 (≥22) N = 573**	***P*-value**
eGFR, (ml/min/1.73m^2), mean ± SD	47.5 ± 19.5	48.1 ± 18.6	47.8 ± 17.7	48.9 ± 17.9	49.7 ± 19	0.035
UACR, (mg/g), median (IQR)	61.8 (327.2)	44.2 (285)	49.5 (331.2)	38.5 (238.5)	34.5 (227.2)	0.032
Urea, (mg/dL), mean ± SD	26.2 (13.6)	25.8 (12.4)	25.3 (13.1)	25.3 (12.1)	23.8 (11.5)	<0.001
Albumin, (g/L), mean ± SD	40.7 ± 4.6	40.9 ± 4.1	41.1 ± 4.2	41.4 ± 4.3	41.1 ± 4.2	0.023
Cholesterol, (mg/dL), mean ± SD	210 ± 49.8	211.6 ± 51.3	218.3 ± 47.3	211.3 ± 50.1	213.9 ± 48.2	0.159
HDL-cholesterol, (mg/dL), mean ± SD	52.7 ± 17.2	55.7 ± 17.2	59.9 ± 19.8	58.3 ± 19.2	62.7 ± 18.8	<0.001
LDL-cholesterol, (mg/dL), mean ± SD	120.6 ± 43.2	120 ± 44.2	125.5 ± 40	119.7 ± 41.5	122.5 ± 40.8	0.440
Triglyceride, (mg/dL), median (IQR)	181 (140)	178 (125)	170 (117.3)	170 (125.5)	149.5 (105)	<0.001
Uric acid, (mg/dL), mean ± SD	7.3 ± 1.9	7.2 ± 1.7	7.1 ± 1.8	7.1 ± 1.7	6.9 ± 1.8	<0.001
Phosphorus, (mmol/L), mean ± SD	1.1 ± 0.3	1.2 ± 0.2	1.2 ± 0.2	1.2 ± 0.2	1.1 ± 0.2	0.350
Calcium, (mmol/L), mean ± SD	2.5 ± 0.2	2.5 ± 0.1	2.5 ± 0.2	2.5 ± 0.2	2.5 ± 0.2	0.095
HbA1c, (%), median (IQR)	5.8 (0.8)	5.8 (0.8)	5.8 (0.8)	5.8 (0.8)	5.8 (0.8)	0.496
CRP, (mg/L), median (IQR)	2.3 (4.2)	2.1 (3.4)	2 (3.8)	2.1 (3.5)	1.7 (3.3)	0.016

Note: Values are expressed as mean values ± standard deviation or medians (interquartile ranges (IRQ)), as appropriate. P-values were obtained by univariate ordinal regression analysis.
Abbreviations: eGFR, estimated glomerular filtration rate based on creatinin (scr); HbA1c, glycated haemoglobin; HDL-cholesterol, high-density lipoprotein-cholesterol; LDL-cholesterol, low-density lipoprotein-cholesterol; UACR, urine albumin-to-creatinine ratio; CRP, C-reactive protein; Q, quintile. Conversion factors for units: calcium in mmol/L to mg/dL, ×4; phosphorus (inorganic) in mmol/L to mg/dL.

Supplementary Table 5. Multivariable adjusted linear regression models for explanation of log-UACR, serum albumin and uric acid by individual components of CKD diet score per 1-SD increase among participants of the German Chronic Kidney Disease (GCKD) observational cohort study (2012-2014).

	Ln UACR			Serum albumin			Uric acid		
**CKD diet score components‡**	β-coefficient	SE	*P*-value	β-coefficient	SE	*P*-value	β-coefficient	SE	*P*-value
Sodium	0.063	0.051	0.220	-0.133	0.109	0.222	-0.056	0.043	0.195
Potassium	-0.013	0.078	0.868	0.293	0.162	0.072	-0.038	0.065	0.556
Fiber	-0.053	0.059	0.369	0.125	0.123	0.310	-0.094	0.049	0.057
Total Protein	0.090	0.077	0.241	-0.057	0.161	0.726	-0.054	0.064	0.402
Sugar	-0.065	0.055	0.234	-0.015	0.114	0.898	-0.024	0.045	0.602
Cholesterol	0.077	0.067	0.253	-0.185	0.142	0.191	0.074	0.056	0.191

UACR concentrations were log transformed due to skewed data, and one unit was added prior to log transformation to avoid loss of data when log(0) is rendered as missing.

‡Each component of CKD diet score was added individually into the model (not adjusted for the other food components), adjusting for gender, age, BMI, caloric intake, smoking, alcohol consumption, school education, and physical activity.
Abbreviations: BMI, body mass index; UACR, urine albumin-to-creatinine ratio; SD, standard deviation.

Supplementary Methods:

Study Design

The study was conducted at the Friedrich-Alexander-University Erlangen-Nuremberg, University Hospital RWTH Aachen, Charité University Berlin, Medical Center University of Freiburg, Medizinische Hochschule Hannover, Medical Faculty of the University of Heidelberg, Friedrich-Schiller-University Jena, Medical Faculty of the LMU Munich, Medical Faculty of the University of Würzburg. At study enrolment, participants (age 18-74 years) were included within the frame of medical care and on the basis of kidney function (estimated glomerular filtration rate (eGFR) 30-60 mL/min/1.73m2, i.e. CKD stage G3, A1-3, or overt proteinuria in the presence of an eGFR > 60 mL/min/1.73m2, i.e. CKD stage G1-2, A3). Overt proteinuria was defined by one of the following criteria: albuminuria > 300mg/g creatinine, albuminuria > 300mg/day, proteinuria > 500mg/g creatinine or proteinuria > 500mg/day. All participants were Caucasian and under constant nephrology care. Exclusion criteria were solid organ or bone marrow transplantation, active malignancy within 24 months prior to screening, New York Heart Association class IV heart failure or unwillingness to provide consent. No particular dietary or nutritional characteristics were considered when inclusion or exclusion criteria were established. Recruitment procedures were neutral with respect to CKD-specific dietary recommendations, though it is possible that patients who participated in the study received nutritional counselling from their treating nephrologist.

The participant's medical and family history, medication and socio-demographic history were recorded by standardized questionnaires. All information was cross-checked by referring to the respective individual's medical records. Anthropometric variables and measurements of resting blood pressure were obtained during physical examination. Diabetes mellitus was defined as HbA1c ≥ 6.5 % or intake of at least one antidiabetic medication.

The ideal body weight (IBW) was calculated using the following equations (1):

$$Men: IBW\ (kg) = 50 + 2.3\ kg * (height\ in\ inch - 60)$$

$$Women: IBW\ (kg) = 45.5\ kg + 2.3\ kg * (height\ in\ inch - 60)$$

IBW is the body weight associated with the lowest mortality for a given height, age, sex and frame size and is based on the Metropolitan life insurance weight-height tables (2). The CKD-specific dietary recommendations use IBW in the anthropometric assessment of adult CKD patients, although the formulae were generated in the general population (3).

Study population for present analysis

The ratio between energy intake and estimated energy requirement was calculated based on present recommended values of the German Society of Nutrition (Deutsche Gesellschaft für Ernährung, DGE) for estimated energy requirement in our cohort, taking into account age, gender and physical activity level.

Measurement of dietary intake

The FFQ captures the average food intake during the past 12 months, including 129 food items and frequency of consumption responses, ranging from “never” to “3 times a day or more frequent”, or up to 11 times a day for beverages, 23 additional questions on preparation methods or fat content and 13 summation questions. From each participant, dietary intake of food groups and derived nutrient intakes (grams per day) were calculated based on standard portion sizes and the German food database (“Bundeslebensmittelschlüssel 2.3”). The intake of supplements was not taken into consideration for the calculation of dietary intake of nutrients.

Health-relevant biomarkers

The biomarkers were: eGFR, urine albumin-to-creatinine ratio (UACR), urea, serum albumin, cholesterol, high-density lipoprotein (HDL) cholesterol, low-density lipoprotein (LDL) cholesterol, triglyceride, calcium, phosphorus (inorganic), uric acid, HbA1c and C-reactive protein (CRP). We have chosen the above-mentioned biomarkers for the following reasons. eGFR and UACR are the two key markers for kidney health as CKD can be classified based on eGFR and UACR. Urea and albumin are valuable for broader nutritional screening and may perceived as biomarkers influenced by dietary protein intake, inflammation and albuminuria. Cholesterol, HDL cholesterol, LDL cholesterol and triglyceride are typical life-style modifiable lipid biomarkers relevant for cardiovascular health. Serum calcium and phosphorus levels can be influenced by diet and are relevant for bone health and other parts of the body. Diet is key to lower uric acid and HbA1c levels, and high values can result in gout and diabetes mellitus type 2, both highly prevalent diseases in patients with CKD (4, 5). Several studies have shown a relation between nutritional status and the inflammatory marker CRP, which plays an important role in the pathogenesis of many chronic diseases (6).

Statistical analysis

Characteristics were age (years, continuous), gender, body mass index (BMI, kg/m^2, continuous), cigarette smoking (smoker, former smoker, never-smoker), alcohol consumption ((regular, >=3 times/week), irregular (<2 times/week) or no consumption of alcoholic beverages), educational attainment (school education <= 9 years, 10 years, >=12 years), physical activity level (<1/week, 1-2x/week, 3-5x/week, >5x/week), systolic blood pressure (mmHg, continuous), diastolic blood pressure (mmHg, continuous), diagnosis of diabetes mellitus (yes, no), and intake of medications: lipid-lowering (yes, no), anti-diabetic (yes, no), anti-hypertensive (yes, no), anti-gout (yes, no). Descriptive statistics was applied to describe dietary intake of sodium, potassium, fiber, total protein, plant-based protein, fat, cholesterol, sugar, carbohydrate and energy intake.

References:

1. Peterson CM, Thomas DM, Blackburn GL, Heymsfield SB. Universal equation for estimating ideal body weight and body weight at any BMI. Am J Clin Nutr. 2016;103(5):1197-203.
2. Shah B, Sucher K, Hollenbeck CB. Comparison of ideal body weight equations and published height-weight tables with body mass index tables for healthy adults in the United States. Nutr Clin Pract. 2006;21(3):312-9.
3. NKF. KDOQI clincal practice guidelines for nutrition in chronic kidney disease: 2019 update. Public review draft october 2019. https://www.kidney.org/professionals/kdoqi-guidelines-commentary-nutrition. Accessed on 4th December 2020.
4. Jing J, Kielstein JT, Schultheiss UT, Sitter T, Titze SI, Schaeffner ES, et al. Prevalence and correlates of gout in a large cohort of patients with chronic kidney disease: the German Chronic Kidney Disease (GCKD) study. Nephrol Dial Transplant. 2015;30(4):613-21.
5. Ko GJ, Kalantar-Zadeh K, Goldstein-Fuchs J, Rhee CM. Dietary Approaches in the Management of Diabetic Patients with Kidney Disease. Nutrients. 2017;9(8).
6. Smidowicz A, Regula J. Effect of nutritional status and dietary patterns on human serum C-reactive protein and interleukin-6 concentrations. Adv Nutr. 2015;6(6):738-47.
